# The Cardioprotective Effects of 4-O-(2″-O-acetyl-6″-O- P-coumaroyl-β-D-glucopyranosyl)-P-coumaric Acid (4-ACGC) on Chronic Heart Failure

**Published:** 2018

**Authors:** Yong Yang, Ke Yu, Yan-min Zhang

**Affiliations:** a *Department of Cardiology, Liaocheng People’s Hospital of Shandong Province, Liaocheng 252000, China. *; b *Department of Emergency, Liaocheng People’s Hospital of Shandong Province, Liaocheng 252000, China.*

**Keywords:** 4-O-(2″-O-acetyl-6″-O- p-coumaroyl-β-D-glucopyranosyl) -p-coumaric acid, Cardioprotective effects, Chronic heart failure, Inflammatory cytokines, Myocardial enzymes

## Abstract

The 4-ACGC isolated from BP was prepared to investigate the cardioprotective effects on attenuating chronic heart failure *in-vivo* and* in-vitro*. A chronic heart failure (CHF) rat model was established to investigate the cardioprotective effects of 4-ACGC. From this, several cardiac function indexes were recorded. The inflammatory markers including tumor necrosis factor-α (TNF-α), Interleukin-6 (IL-6), and Interleukin-1β (IL-1β) were evaluated by enzyme-linked immunosorbent assay (ELISA). Subsequently, serum levels of myocardial enzymes lactate dehydrogenase (LDH) and creatine kinase (CK) were also assessed by ELISA kits. Ultimately, the cardioprotective and anti-inflammatory effects of 4-ACGC were further verified on CMECs. The results showed that the treatment of 4-ACGC significantly reduced cardiac hypertrophy and reversed the Ejection Fraction (EF), Heart Rate (HR), Fractional Shortening (FS), and Cardiac Output (CO) changes in CHF rats. The treatment of 4-ACGC could effectively inhibit the inflammatory cytokines induced by CHF. It’s also showed that a reverse effect of 4-ACGC on serum increased levels of LDH and CK in CHF rats. The increased levels of IL-1β and IL-6 stimulated by TNF-α on CMECs were also decreased after treating with 4-ACGC. The present study provided experimental evidence that 4-ACGC possessed obvious cardioprotective effects on attenuating CHF. 4-ACGC could suppress the expression of inflammatory mediators and myocardial enzymes, which might be one of the mechanisms.

## Introduction

It was well known that chronic heart failure (CHF) is a complex condition with structural abnormalities, functional disorders or a combination of them. It could impair the heart ability to function as a pump to support physiological circulation (1). CHF is reported to be the ultimate clinical manifestation of various cardiovascular diseases, and it is also one of the leading causes of disability and deaths in patients with cardiovascular disease worldwide (2-3). The existing drugs, such as β-blockers, angiotensin receptor antagonists and mineralocorticoid receptor blockers were used to treat patients with CHF (4-6), but they have severe adverse effects and cannot fully prevent progressive cardiac remodeling and dysfunction. Therefore, finding optimal drug for the therapy of heart failure is still a considerable challenge. 

*Bidens pilosa* Linn. (BP), belonging to Asteracea family, is widely found in tropical and subtropical areas worldwide, has been used to treat diseases including stomach illnesses, malaria infections and liver disorders (7-9). BP was used in Traditional Chinese Medicine for the treatment of peri-appendicular abscess, and it was a major ingredient of herbal tea in Taiwan to prevent inflammation and cancer (10-11). Previous researches have reported constituents isolated and identified from this plant possessed a lot pharmacological activities including anti-inflammatory, anti-cancer, immunosuppressive, antibacterial, and anti-hyperglycemic effects, etc (11-14). However, to the best of our knowledge, few researches have reported the cardioprotective effects activity of the monomers from this plant.

**Figure 1:**
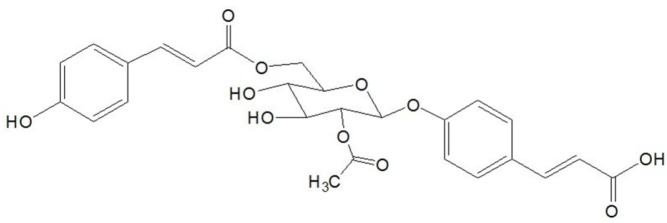
Structure of 4-ACGC

In the present study, a CHF rat model was used to investigate the cardioprotective effects of 4-ACGC (Figure 1). Several cardiac function indexes and the relationship between 4-ACGC treatment and inflammatory cytokines were assessed. Subsequently, lactate dehydrogenase (LDH) and creatine kinase (CK) were also assayed. Ultimately, the cardioprotective and anti-inflammatory effects of 4-ACGC were further verified on CMECs.

## Experimental

*Chemicals and Reagents*

Tumor necrosis factor-α (TNF-α), Interleukin-6 (IL-6), and Interleukin-1β enzyme-linked immunosorbent assay (ELISA) kits were purchased from Pierce/Endogen Co. (Rockford, USA). Human TNF-α was obtained from Pepro Tech (Rocky Hill, NJ). Creatine kinase (CK) and lactate dehydrogenase (LDH) ELISA kits were from R&D systems (Beijing, China). Pentobarbital was purchased from Beijing propbs Biotechnology Co., Ltd (Beijing, China). All other chemicals and reagents used in the study were of analytical reagent grade.

*Separation and purification of 4-ACGC from BP*

The whole plant of BP was collected from Guangdong Province, and identified by an expert in the Traditional Chinese Medicine (TCM) department of our hospital. A voucher specimen was deposited in the laboratory herbarium of Liaocheng People’s Hospital of Shandong Province (T20140912).

The dried and powered BP was extracted with 60% EtOH by reflux for three times (2 h for each extraction). Then, the EtOH extract was partitioned with n-butanol, ethyl acetate, and petroleum ether, respectively. A residue of the ethyl acetate fraction was obtained under reduced pressure at 50 °C with a vacuum rotary evaporator.

The ethyl acetate fraction was eluted through silica-gel (100-200 mesh) with petroleum ether: ethyl acetate (15:1, 10:1, 8:1, 5:1, 3:1, and 1:1) and a series of subfractions (I-VI) were obtained. Subsequently, a series of chromatographic techniques including columns of silica gel (200-300 mesh) and Sephadex LH-20 were used to purify 4-ACGC. As a result, purified product was isolated from fractions V.

*Identification of 4-ACGC*

The 4-ACGC was identified by ^1^H-NMR and ^13^C-NMR, and the results were compared with the previous reference (14, 15). Besides, the HPLC was used to evaluate its purity, and the results showed that purity of the 4-ACGC was not less than 98%. The ^1^H-NMR and ^13^C-NMR spectrum results are as follows:

C_26_H_26_O_11_; ^1^H-NMR (600 MHz, DMSO-d6) δ (ppm): 7.64 (2H, d, J = 8.2 Hz, H-2, 2′), 7.60 (2H, d, J = 8.8 Hz, H-6, 6′), 7.59 (2H, d, J = 15.8 Hz, H-7), 7.57 (2H, d, J = 15.8 Hz, H-7′), 7.53 (2H, d, J = 8.8 Hz, H-5, 5′), 7.40 (2H, d, J = 8.2 Hz, H-3, 3′), 6.77 (2H, d, J = 16.4 Hz, H-8), 6.47 (2H, d, J = 16.4 Hz, H-8′), 5.62 (1H,d, J = 8.6 Hz, H-1″), 5.57 (1H, dd, J = 8.6, 9.3 Hz, H-2″), 4.92 (1H, dd, J = 3.0, 11.2 Hz, H-6a″), 4.81 (1H, dd, J = 6.2, 11.2 Hz, H-6b″), 4.51 (1H, m, H-3″), 4.29 (1H, m, H-4″), 4.22 (1H, m, H-5″), 2.14 (3H, s, -OCOCH3); 13C-NMR (150 MHz, DMSO-d6) δ (ppm): 170.2 (C-9), 169.5, (-OCOCH3), 168.3 (C-9′), 161.0 (C-4), 158.5 (C-4′), 145.3 (C-7), 144.3 (C-7′), 134.3 (C-1), 134.2 (C-2 and 2′), 129.1 (C-6 and 6′), 128.0 (C-1′), 119.1 (C-8),118.3 (C-3 and 3′), 117.1 (C-5 and 5′), 114.7 (C-8′), 98.4 (C-1″), 76.3 (C-3″), 73.5 (C-2″), 73.3 (C-5″), 73.0 (C-4″), 65.3 (C-6″).

*Animals*

Male Sprague-Dawley rats weighing 225 ± 14g were housed under controlled conditions in a quarantine room and maintained with free access to food and water under a 12 h light-dark cycle. The animals were feeding for a week acclimatization period before experiments. All of the experiments done in this study were approved by the Animal Ethics Committee of Liaocheng People’s Hospital of Shandong Province.

*Experimental protocols*

The rats were anaesthetized by intraperitoneal injection of pentobarbital (40 mg/kg). Myocardial infarction and heart failure model were established according to the methods as previously described (16, 17). In brief, left thoracotomy was carried out under sterile conditions to open the pericardium. The heart was exteriorized and the left anterior descending coronary artery was ligated using 6-0 suture approximately 2-3 mm distal from its origin. Then the heart was replaced into the chest and the thorax was closed. The survived rats were randomly divided into five groups (n = 14) the sham group, the CHF group, and the low-dose 4-ACGC group (15 mg/kg/d), the medium-dose 4-ACGC group (30 mg/kg/d), the high-dose 4-ACGC group (60 mg/kg/d). For the sham group, the rats were given the same operation without ligation of the left coronary artery. The treatment by intragastric administration was continued for eight weeks. After that, the cardiac function of all groups was examined. The blood samples were drawn from the abdominal aorta and the serum was stored at -80 °C before assaying. 

*Cell culture*

Rat cardiac microvascular endothelial cells (CMECs) were isolated from hearts of SD rats (1-3 days old) as previously described (18). The CMECs were isolated from heart tissues by 0.2% collagenase type II and 0.1% trypsin, and incubated for 35 min at 37 °C in a shaking water bath. The solution was filtered and centrifuged (1000 g, 10 min), and the cells were resuspended in DMEM/F12 with 10% fetal bovine serum (FBS). Unattached cells and debris were washed off after 90 min. Cultured cells formed confluent monolayers within 5 to 7 days and the CMECs were starved for one day in DMEM with 1% FBS before pharmacologic treatments.

*Determination of cardiac function*

The rats were anesthetized intraperitoneally with pentobarbital (40mg/kg). A Vivid I handled ultrasound (GE Healthcare) equipped with a 12-MHz linear transducer was used to assay the Ejection Fraction (EF), Heart Rate (HR), Fractional Shortening (FS) and Cardiac Output (CO) of the rats, respectively. 

*Determination of levels of TNF-α, IL-6 and IL-β in heart tissues*

The myocardial tissues were homogenized in the RIPA lysis buffer (1:10, v/v) and then centrifuged (6000 g, 4 °C and 15 min). TNF-α, IL-6 and IL-β were analyzed by enzymelinked immunosorbent assay (ELISA), using kits according to the manufacturer instructions.

*Determination of LDH and CK in serum*

Serum was separated from whole blood using high-speed centrifugal (6000 g, 4 °C and 15 min). Serum levels of myocardial enzymes LDH and CK were measured using ELISA kits according to the manufacturer instructions.

*CMEC inflammation model and determination of IL-1β and IL-6*

CMEC cells were cultured in 96-well plates and 4-ACGC (0.2, 0.4, 0.8 mg/mL) were added to the wells for 24 h within TNF-α (100 ng/mL). The levels of IL-1β and IL-6 in the cell culture supernatant were measured using ELISA kits according to the manufacturer instructions. 

*Statistical analysis*

Data analysis was performed using SPSS 13.0 software and all data were expressed as mean ± S.D. Statistical evaluation was carried out using One-way ANOVA method followed by Tukey›s multiple comparison test. The *P* value less than 0.05 was accepted as statistically significant.

## Results

*Effects of 4-ACGC on body weight and cardiac weight*

As can be seen from Table 1, the whole heart weight (*P* < 0.05), left heart weight (*P* < 0.01), index of heart/body weight (*P* < 0.05) and index of left heart/body weight (*P* < 0.05) were significantly increased in CHF rats. However, the body weight of the CHF groups decreased significantly (*P* < 0.01) compared with Sham group.

The body weight of 4-ACGC treated groups was increased significantly at the concentrations of 30 mg/kg (*P* < 0.05) and 60 mg/kg (*P* < 0.01) compared with CHF groups. The whole heart weight of 4-ACGC treated groups was decreased significantly at the doses of 30 mg/kg and 60 mg/kg (*P* < 0.01) compared with CHF groups. The left heart weight of 4-ACGC treated groups were decreased significantly at the doses of 15 mg/kg (*P* < 0.05), 30 mg/kg (*P* < 0.05) and 60 mg/kg (*P* < 0.01) compared with CHF groups. The index of heart/body of 4-ACGC treated groups wasdecreased significantly at the doses of 15 mg/kg, 30 mg/kg and 60 mg/kg (p < 0.05) compared with CHF groups. The index of left heart/body of 4-ACGC treated groups was decreased significantly at the doses of 30 mg/kg and 60 mg/kg (*P* < 0.05) compared with CHF groups. All these indicated that 4-ACGC could reduce cardiac hypertrophy in CHF. 

*Effects of 4-ACGC on cardiac function in CHF rats*

The effects of 4-ACGC on cardiac function in CHF rats were shown in Table 2. The Compared with the sham groups, the Fractional Shortening (FS) (*P* < 0.01), Ejection Fraction (EF) (*P* < 0.01), Cardiac Output (CO) (*P* < 0.0s5) and Heart rates (HR) (*P* < 0.01) were significantly decreased in CHF groups. However, the FS and EF of 4-ACGC treated groups showed significant increase at the doses of 30 mg/kg (*P* < 0.05) and 60 mg/kg (*P* < 0.01). The CO of 4-ACGC treated groups showed significant increase at the doses of 15 mg/kg, 30 mg/kg and 60 mg/kg (*P* < 0.05). The HR of 4-ACGC treated groups showed significant increase at the doses of 15 mg/kg (*P* < 0.05), 30 mg/kg (*P* < 0.05) and 60 mg/kg (*P* < 0.01). The results showed that the treatment of a-AGCC can significantly reverse the FS, EF, CO and HR variables in CHF rats.

*Effects of 4-ACGC on TNF-α, IL-1β and IL-6 levels in CHF rats*

**Figure 2:**
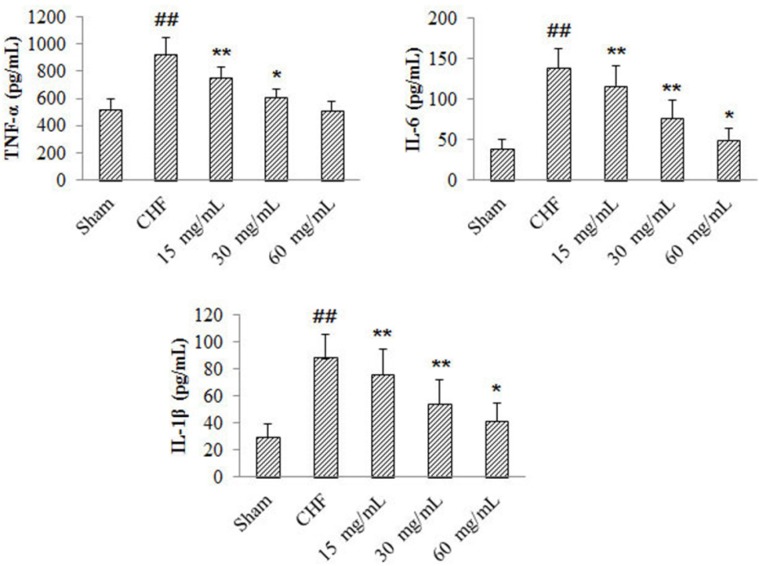
Effects of 4-ACGC on TNF-α, IL-1β and IL-6 in CHF rats. Data were expressed as Mean ± S.D. ^##^*P* < 0.01, compared with sham group. *P < 0.05, compared with CHF group. **P < 0.01, compared with CHF group

Compared with sham group, myocardial production of TNF-α, IL-1β and IL-6 in CHF group increased significantly (*P* < 0.01) (Figure 2). The TNF-α levels of 4-ACGC treated groups decreased significantly at the doses of 15 mg/kg (*P* < 0.01) and 30 mg/kg (*P* < 0.05) compared with the CHF group. The IL-1β and IL-6 levels of 4-ACGC treated groups decreased significantly at the doses of 15 mg/kg (*P* < 0.01), 30 mg/kg (*P* < 0.01) and 60 mg/kg (*P* < 0.05) compared with the CHF group. The results indicated a close relationship between CHF and inflammation, and the treatment of 4-ACGC could effectively inhibit the inflammatory cytokines in CHF rats.

*Effect of 4-ACGC on LDH and CK in CHF rats*

**Figure 3:**
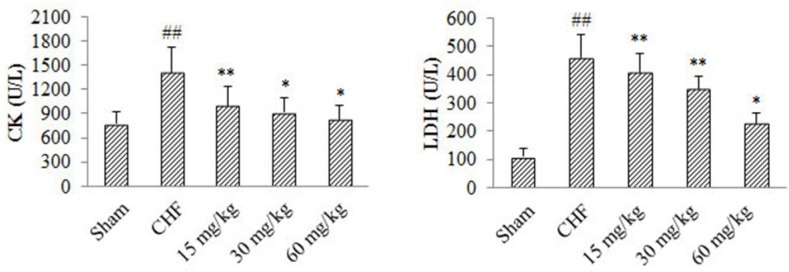
Effects of 4-ACGC on CK and LDH in CHF rats. Data were expressed as Mean ± S.D. ^##^*P* < 0.01, compared with sham group. *P < 0.05, compared with CHF group. **P < 0.01, compared with CHF group

As shown in figure 3, compared with Sham group, serum levels of LDH and CK in CHF group increased significantly (*P* < 0.01). Serum CK level of 4-ACGC treated groups at the doses of 15 mg/kg (*P* < 0.01), 30 mg/kg *P* < 0.05) and 60 mg/kg (*P* < 0.05) showed significant decrease compared with CHF group. Serum LDH level of 4-ACGC treated groups at the doses of 15 mg/kg (*P* < 0.01), 30 mg/kg (*P* < 0.01) and 60 mg/kg (*P* < 0.05) showed significant decrease compared with CHF group. The results indicated a reverse effect of 4-ACGC on serum levels of LDH and CK in CHF rats.

*Effects of 4-ACGC on IL-1β and IL-6 levels on CMECs*

**Figure 4:**
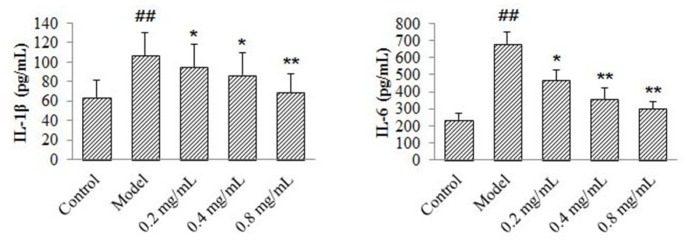
Effects of 4-ACGC on IL-1β and IL-6 expression on TNF-α-induced CMECs. Data were expressed as Mean ± S.D. ^##^*P* < 0.01, compared with sham group. *P < 0.05, compared with CHF group. **P < 0.01, compared with CHF group

According to the results in figure 4, compared with control group, production of IL-1β and IL-6 in the model group increased significantly (*P *< 0.01). The IL-6 levels of 4-ACGC treated groups decreased significantly at the concentrations of 0.2 mg/kg (*P* < 0.05), 0.4 mg/kg (*P* < 0.01) and 0.8 mg/kg (*P* < 0.01) compared with the model group. The IL-1β levels of 4-ACGC treated groups decreased significantly at the concentrations of 0.2 mg/kg (*P* < 0.05), 0.4 mg/kg (*P* < 0.05) and 0.8 mg/kg (*P* < 0.01) compared with the model group. The results confirmed the protective and anti-inflammatory effects of 4-ACGC on CMECs.

## Discussion

To the best of our knowledge, the present study was the first report regarding the cardioprotective effects of 4-ACGC on chronic heart failure *in-vivo *and* in-vitro*. The 4-ACGC showed obvious cardioprotective effects *in- vivo *and* in-vitro* by suppressing the expression of inflammatory mediators and myocardial enzymes.

**Table 1:** Effects of 4-ACGC on body weight and cardiac weight

	**Body Weight** **(g)**	**Whole heart** **Weight (g)**	**Left heart** **Weight (g)**	**Index of** **Heart/body**	**Index of left** **Heart/body**
Sham	301.06 ± 14.18	0.92 ± 0.13	0.53 ± 0.07	0.32 ± 0.06	0.18 ± 0.05
CHF	273.32 ± 10.05 ##	1.11 ± 0.11 #	0.81 ± 0.12 ##	0.42 ± 0.07 #	0.29 ± 0.06 #
15 mg/kg	275.02 ± 10.31	1.06 ± 0.15	0.69 ± 0.07 *	0.35 ± 0.08 *	0.28 ± 0.05
30 mg/kg	292.63 ± 10.42 *	0.98 ± 0.11 *	0.62 ± 0.08 *	0.35 ± 0.06 *	0.23 ± 0.05 *
60 mg/kg	298.36 ± 8.15 **	0.91 ± 0.10 *	0.54 ± 0.08 **	0.31 ± 0.06 *	0.21 ± 0.06 *

Data were expressed as Mean ± S.D.

# *P* < 0.05, compared with sham group.

## *P *< 0.01, compared with sham group.

* *P* < 0.05, compared with CHF group.

** *P* < 0.01, compared with CHF group.

**Table 2:** Effects of 4-ACGC on FS, EF, CO and HR in CHF rats

	**FS (%)**	**EF (%)**	**CO (L/min)**	**HR (BPM)**
Sham	46.71 ± 8.15	78.31 ± 13.57	0.21 ± 0.06	381 ± 41
CHF	32.84 ± 6.07 ##	56.19 ± 10.41 ##	0.11 ± 0.05 #	287 ± 47 ##
15 mg/kg	35.31 ± 8.27	61.76 ± 13.75	0.16 ± 0.06 *	312 ± 36 *
30 mg/kg	39.03 ± 7.63 *	69.64 ± 12.65 *	0.17 ± 0.07 *	347 ± 42 *
60 mg/kg	44.96 ± 8.93 **	71.51 ± 12.52 **	0.20 ± 0.07 *	377 ± 43 **

Data were expressed as Mean ± S.D.

# *P* < 0.05, compared with sham group.

## *P* < 0.01, compared with sham group.

* *P* < 0.05, compared with CHF group.

** *P* < 0.01, compared with CHF group.

It was reported that pro-inflammatory cytokines played important roles in the ischemic heart diseases. In both experimental animal models and patients, myocardial fibrosis and the onset and progression of cardiac remodeling were closely associated with the induction of pro-inflammatory cytokines (19, 20). TNF-α, IL-1β and IL-6 were the main pro-inflammatory cytokines identified as contributors to the syndrome of chronic heart failure (21, 22). So, the relevant cytokines of CHF rats were determined in the present study and the results showed that the cytokines were increased significantly in CHF model groups. However, the treatment of 4-ACGC could effectively inhibit the TNF-α, IL-1β and IL-6 induced by CHF. Therefore, we believed that the protective effects of 4-ACGC could largely be a result of the suppression of the inflammatory response by the inhibition of pro-inflammatory mediators.

Myocardium possesses high abundance of enzymes like creatine kinase (CK) and lactate dehydrogenase (LDH) and the enzymes serve as sensitive indexes to assess the severity of myocardial infarction (23). The levels of the two enzymes were evaluated in the present study. They were increased significantly in the serum of CHF rats, however, they were decreased after treating with 4-ACGC. The results indicated that 4-ACGC could prevent cardiac damage by decreasing the marker enzymes.

The cardioprotective and anti-inflammatory effects of 4-ACGC were further verified on CMECs. TNF-α were used to stimulate signal transduction to increase inflammatory cytokines (24). The release of IL-6 and IL-1β on CMECs after drug and TNF-stimulation was measured. As a result, the levels of IL-1β and IL-6 were decreased after treating with 4-ACGC. The results confirmed the protective and anti-inflammatory effects of 4-ACGC.

In addition, it was demonstrated that 4-ACGC had significant cardioprotective effects, indicating that 4-ACGC and its derived compounds might be the substance basis of BP for anti-inflammatory and cardioprotective effects by orally administration. However, more researches could be taken to investigate systemically the cardioprotective effects of 4-ACGC and its derives by orally administration in the future.

## Conclusion

In summary, the present study provided experimental evidence that 4-ACGC possessed obvious cardioprotective effects on CHF. 4-ACGC could suppress the expression of inflammatory mediators and myocardial enzymes, which might be one of the mechanisms. 
